# Real-Life Application of the Penicillin Allergy Decision Rule (PEN-FAST) Score in De-Labeling Penicillin Allergy: A Case Series

**DOI:** 10.7759/cureus.114245

**Published:** 2026-08-10

**Authors:** Fahmida Begum, Takudzwa J Dhlandhlara, Nadia Qazi, Aylin Ugurlu, Rahul Mukherjee

**Affiliations:** 1 Respiratory Medicine, Heartlands Hospital, Birmingham, GBR; 2 Nephrology, Heartlands Hospital, Birmingham, GBR; 3 Respiratory Medicine, University Hospitals Birmingham NHS Foundation Trust, Birmingham, GBR

**Keywords:** allergy risk stratification, antimicrobial resistance, hypersensitivity reactions, pen-fast, penicillin allergy

## Abstract

Penicillin allergies are common but frequently inaccurate, resulting in unnecessary use of broad-spectrum antibiotics and contributing to antibiotic resistance. Tools such as the Penicillin Allergy Decision Rule (PEN-FAST) support the assessment of penicillin allergy risk and can help identify low-risk individuals who may be appropriate candidates for a direct oral penicillin challenge. We report four cases of patients hospitalized with respiratory infections, including infective exacerbation of bronchiectasis, bronchopneumonia, and empyema thoracis, all with documented penicillin allergy labels. In each case, a more detailed review of the allergy history revealed reactions inconsistent with IgE-mediated hypersensitivity reactions, including isolated diarrhea, leg swelling, and delayed rashes. All patients had a PEN-FAST score of 0, which indicated a low risk of a true penicillin allergy. These patients underwent supervised direct oral challenges with amoxicillin or co-amoxiclav, which revealed no adverse reactions. Following a successful oral challenge, penicillin allergies were de-labeled, and the patients were started on optimal antibiotic treatment. All cases revealed favorable outcomes, such as improvements in inflammatory markers and symptoms.

## Introduction

Penicillin allergies are frequently reported, yet many patients tolerate penicillin after additional assessment [1]. Around 10% of patients in the UK have a “penicillin allergy label” recorded, with 90% of these labels being inaccurate [2]. Most of these diagnoses are made in childhood, relating to events that do not indicate a true penicillin allergy, including isolated diarrhea, nausea, and vomiting [3].

The term “allergy” may be used inaccurately to refer to adverse reactions that are not immunologically mediated [3]. Inappropriate labeling of penicillin allergy can lead to the use of broad-spectrum antibiotics, antimicrobial resistance, and treatment failure [1]. A penicillin allergy includes immediate IgE-mediated reactions, such as urticaria, angioedema, and anaphylaxis, as well as delayed T-cell-mediated reactions, including severe cutaneous adverse reactions [1].

Skin rashes are the most frequent trigger for allergy labeling in children [3]. Viral infections and drug-virus reactions frequently cause childhood rashes, which are often mislabeled as penicillin allergies [3]. Clinical history, with tests such as skin testing and drug challenges, is essential for an accurate diagnosis of a true penicillin allergy [1].

The Penicillin Allergy Clinical Decision Rule (PALACE) trial assessed whether direct oral penicillin challenge was as safe and effective as penicillin skin testing followed by oral challenge in adults with a low-risk penicillin allergy (PEN-FAST score <3) [4]. This randomized trial of 382 patients showed no added benefit from skin testing and similar de-labeling rates [4]. This supports direct oral challenge as a faster, simpler alternative and validates PEN-FAST for risk stratification, suggesting that skin testing is no longer required as the standard in low-risk individuals [4].

PEN-FAST is a clinical decision tool to identify patients at low risk of a true penicillin allergy who are suitable for direct oral challenge without prior skin testing [5]. Thus, the PEN-FAST tool complements the de-labeling pathway by guiding who can safely proceed to the drug provocation test, which remains the diagnostic gold standard [5]. This clinical decision rule has four questions, adding up to a total score ranging from 0 to 5 [1]: reaction occurred five years ago or less (F), anaphylaxis or angioedema (A), severe cutaneous adverse reaction (S), and treatment required for the reaction (T). Scores of 0-2 are associated with a low risk of true penicillin allergy, scores of 3 are associated with moderate risk, and scores of 4-5 are associated with high risk [6].

This case series explores the identification and de-labeling of patients with incorrect penicillin allergy labels, highlighting the role of risk stratification, assessment, and direct oral challenge in safely removing inaccurate allergy labels and facilitating access to optimal antibiotics.

## Case presentation

Case 1 

A 70-year-old female was admitted with confusion, cough, and chest pain. She was also reported to have a productive cough with increased purulent sputum. She had a background of bronchiectasis, ischemic heart disease, pulmonary fibrosis, type 2 diabetes mellitus, and rheumatoid arthritis.

On admission, she had a low-grade fever of 37.4°C, with oxygen saturation (SpO₂) of 94% on room air, falling to 91% while speaking. Baseline laboratory investigations are summarized in Table 1. Viral swabs were taken and tested positive for human metapneumovirus.

**Table 1:** Summary of laboratory findings.

Case	Diagnosis	PEN-FAST score	Oral drug challenge	CRP (mg/L) on admission (reference range: <5 mg/L)	White cell count (× 10⁹/L) on admission (reference range: 4.0-10.0 × 10⁹/L)	CRP (mg/L) after treatment with optimal antibiotic (reference range: <5 mg/L)	White cell count (× 10⁹/L) after treatment with optimal antibiotic (reference range: 4.0-10.0 × 10⁹/L)
1	Infective exacerbation of bronchiectasis	0	625 mg co-amoxiclav	167	10.99	60	6.6
2	Infective exacerbation of bronchiectasis with bronchopneumonia	0	625 mg co-amoxiclav	211	16.75	42	5.8
3	Bronchopneumonia	0	500 mg amoxicillin	237	15.55	30	9.46
4	Right-sided empyema thoracis	0	500 mg amoxicillin	175	15.23	58	5.24

She was treated for an infective exacerbation of bronchiectasis on a background of nonspecific interstitial pneumonia and rheumatoid arthritis. She had a documented penicillin allergy label and was therefore initially commenced on ertapenem.

On further assessment of her allergy history, the patient was unsure of the type of reaction she had experienced following penicillin exposure. Using the PEN-FAST tool, she was further assessed and had a score of 0, indicating a low risk of a true penicillin allergy. An oral challenge was discussed with the patient, and she agreed. A supervised oral challenge with co-amoxiclav was undertaken using a single 625-mg dose of co-amoxiclav. The patient was observed regularly for four hours following administration of the drug for any acute reactions. No adverse features developed during the observation period, and the challenge was considered negative. Following this negative challenge, her penicillin allergy was de-labeled. She was then switched to oral co-amoxiclav for 24 hours and was switched from IV ertapenem to IV co-amoxiclav, which was tolerated well.

Case 2 

A 76-year-old male presented with fever, shortness of breath, and a productive cough on a background of bronchiectasis. Initial laboratory investigations demonstrated markedly elevated inflammatory markers (Table 1).

During his hospital admission, he was treated for an infective exacerbation of bronchiectasis and bronchopneumonia. A penicillin allergy was documented in his records. He was therefore started on levofloxacin. On further clarification of his allergy, however, the patient reported isolated leg swelling after previous penicillin exposure, without any immediate hypersensitivity reactions such as angioedema, urticaria, tongue swelling, or respiratory compromise.

Assessment using the PEN-FAST tool showed a score of 0. A supervised oral challenge with a single 625-mg dose of co-amoxiclav was therefore administered after further discussion and agreement with the patient. The patient was then observed for 4 hours and tolerated this well, with no acute reactions. The patient was then switched from levofloxacin to co-amoxiclav and completed a 10-day course of co-amoxiclav.

Following treatment with co-amoxiclav, inflammatory markers improved significantly, with CRP and white cell count trending downward (Table 1). The patient was successfully de-labeled as penicillin-allergic during this admission following a negative challenge and tolerated co-amoxiclav well.

Case 3 

An 83-year-old female was admitted with a one-week history of worsening shortness of breath, sore throat, and a productive purulent cough. She was pyrexial on presentation. She had a previous diagnosis of chronic obstructive pulmonary disease, which had subsequently been de-labeled.

Initial laboratory investigations are summarized in Table 1. Extended viral swabs were negative. Her presentation and further investigations were consistent with bronchopneumonia. She had a documented penicillin allergy; however, on further clarification, her reported reaction had occurred many years previously and consisted of an isolated episode of diarrhea. She denied any features suggestive of a true allergy, including urticaria, angioedema, or breathlessness.

Given her PEN-FAST score of 0, she underwent a supervised oral challenge with a single 500-mg dose of amoxicillin, which was tolerated well with regular observations. No adverse reactions occurred following the oral challenge. Subsequently, the penicillin allergy was successfully de-labeled, and the patient was started on IV amoxicillin and regular nebulizers.

The patient completed a five-day course of amoxicillin, with a good clinical response characterized by resolution of pyrexia and improvement in inflammatory markers and respiratory symptoms.

Case 4 

A 67-year-old female was admitted with a three-month history of progressive respiratory symptoms and recurrent lower respiratory tract infections. During this period, she had two microbiologically confirmed episodes of infection with *Streptococcus pneumoniae*.

**Figure 1:**
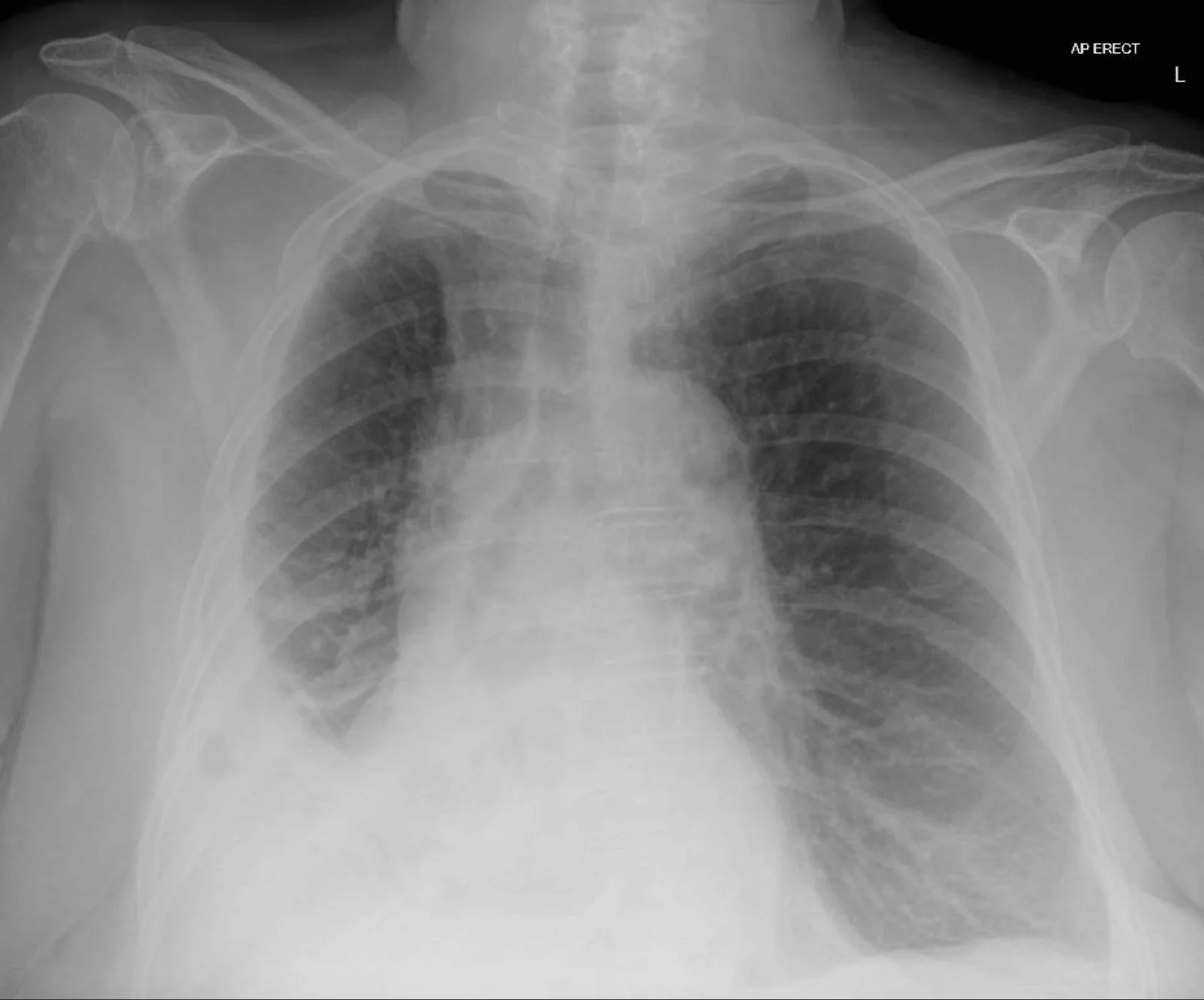
Chest radiograph showing right-sided lower-zone opacification, demonstrating a right-sided pleural collection representative of right-sided empyema thoracis.

On this admission, she presented with worsening breathlessness and malaise. A bedside thoracic ultrasound was performed, which identified a significant loculated effusion extending across three intercostal spaces on the right side. Imaging, including chest radiography, demonstrated a right-sided pleural collection (Figure 1). The pleural aspirate was sent for microbiology, which grew *Streptococcus anginosus*. She maintained oxygen saturation (SpO₂) of 94% while receiving 28% oxygen via a Venturi mask. The clinical diagnosis was right-sided empyema thoracis, likely secondary to *S. anginosus*.

This patient had a documented penicillin allergy. On taking a more detailed allergy history, she reported a delayed-onset rash that occurred during pregnancy many years ago. The rash occurred several days after penicillin exposure. It consisted of a small, localized erythematous patch with a few vesicles and mild pruritus. The rash was self-limiting and required no medical management. She denied any features associated with anaphylaxis or angioedema.

Her PEN-FAST score was 0. Following counseling regarding the risks and benefits, the patient consented to a supervised oral amoxicillin challenge. A 500-mg dose of amoxicillin was administered, and the patient was observed for 4 hours. The challenge was completed without any adverse reactions. The penicillin allergy label was subsequently removed, allowing access to first-line beta-lactam therapy for the management of her right-sided empyema.

## Discussion

These cases demonstrate the value of structured penicillin allergy assessment using the PEN-FAST clinical decision tool. All four patients had low-risk historical penicillin allergy labels (PEN-FAST score 0) and successfully underwent supervised oral amoxicillin or co-amoxiclav challenges without adverse reactions. De-labeling enabled the use of first-line beta-lactam therapy.

This facilitated de-escalation from broad-spectrum antibiotics such as ertapenem and levofloxacin, supporting antimicrobial stewardship. All patients showed favorable clinical outcomes following beta-lactam treatment. These cases highlight the importance of detailed allergy assessments and demonstrate that penicillin allergy de-labeling can safely and effectively be completed in carefully selected low-risk inpatients. These findings reinforce the recognized issue of over-reporting penicillin allergies, where historical labels are frequently based on nonspecific reactions. In our cases, reported symptoms included isolated gastrointestinal upset and peripheral swelling, which were not consistent with immediate hypersensitivity reactions. This highlights the need for careful re-evaluation rather than reliance on previous documentation alone.

Incorrect labeling results in the use of broad-spectrum antibiotics. Although not reported in the four cases, it is important to note the risks associated with the use of broad-spectrum antibiotics, including *Clostridioides difficile*, methicillin-resistant *Staphylococcus aureus*, and vancomycin-resistant *Enterococci *infections [6]. Alongside this, it has been identified that penicillin allergy labels have resulted in increased hospital stays due to treatment failures secondary to the use of less effective alternative antibiotics [3].

The PEN-FAST clinical decision tool provided a validated approach to risk stratification, correctly identifying all patients as suitable for direct oral challenge without preceding formal skin testing. This aligns with contemporary evidence, including the PALACE trial [4], which demonstrated that direct oral penicillin challenge in adults with low-risk allergy was as safe and efficient as the two-step approach of skin testing followed by oral challenge. Collectively, these data support a shift away from routine skin testing in low-risk patients.

Beyond individual patient benefit, the case series demonstrates a wider system-level benefit of de-labeling penicillin allergies for inpatients, such as cost-effectiveness. Alternative antimicrobials are more expensive than those used when a patient is able to tolerate penicillin [3]. Picard et al. conducted a retrospective study that demonstrated that reported penicillin allergies were associated with increased expenditure over a year, largely due to the use of non-beta-lactam antibiotics [7].

Although this case series included a small sample size in a single center, much of the research and data highlight the importance of implementing PEN-FAST to support antimicrobial stewardship. Antibiotic stewardship is of great importance in today’s clinical practice in view of antimicrobial resistance. Moreover, in many clinical scenarios, penicillins are the superior choice, and for such patients, de-labeling of penicillin allergy is highly beneficial, further underlining the importance of de-labeling. This case series confirms the real-life effectiveness of the PEN-FAST score in de-labeling incorrect labels in four consecutive patients on the respiratory support unit, thereby strengthening the need for regular use of PEN-FAST scoring.

## Conclusions

This case series demonstrates that many hospitalized patients carrying penicillin allergy labels are suitable for reassessment using the PEN-FAST clinical decision rule. In patients identified as low risk, supervised direct oral challenge can safely facilitate penicillin allergy de-labeling, allowing access to first-line beta-lactam therapy and supporting antimicrobial stewardship. Incorporation of a structured allergy assessment into routine inpatient care may reduce unnecessary broad-spectrum antibiotic use and improve infection management outcomes.

The absence of post-discharge follow-up in this case series, however, represents a limitation, as longer-term follow-up would provide additional information regarding the long-term success of de-labeling. Larger studies are also needed to further validate these findings and support the broader implementation of the PEN-FAST score in helping de-label incorrect penicillin allergy labels in routine clinical practice.
